# Supplementary material for: A new statistical framework for genetic pleiotropic analysis of high dimensional phenotype data
Source: BMC Genomics. 2016 Nov 7;17:881. doi: 10.1186/s12864-016-3169-1 (PMC5100198; doi:10.1186/s12864-016-3169-1)
Supplement: Additional file 1: Figure S1. — A scheme of genotype-phenotype network. Figure S2. Diagram associated with effect decomposition. Figure S3. Diagram of a simulation example for illustrating equation (12). Figure S4. An example for the simulated genotype-phenotype network. The network consisted of ten phenotype nodes and 30 genotype (SNP) nodes. Figure S5. Performance of S2SEM and SML for phenotype network inference. The power and FDR of the two methods for inference of phenotype networks when the phenotype and genotype number is 10 and 30 respectively. Figure S6. A genotype-phenotype network consisted of two genes that were reported to be associated with phenotypes in the analysis or other CVD related phenotypes in the literatures and ten phenotypes (one isolated phenotype didn’t appear) estimated using QTLnet method. The nodes in yellow color represented the phenotypes, the nodes in the red color represented genes, the black arrows indicated the causal relations between phenotypes and the blue arrows indicted the contribution of the gene to one phenotype. (DOCX 4101 kb) [file 12864_2016_3169_MOESM1_ESM.docx]

**
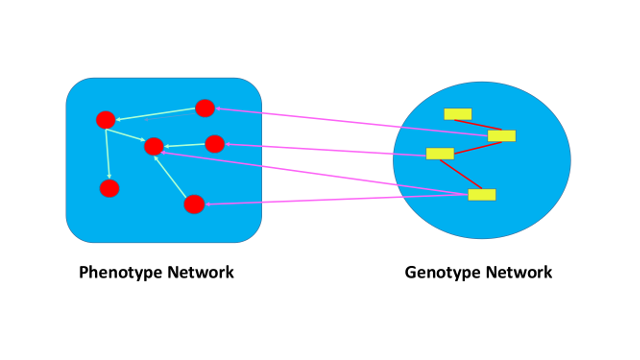
**

**Figure S1.** A scheme of genotype-phenotype network.

**
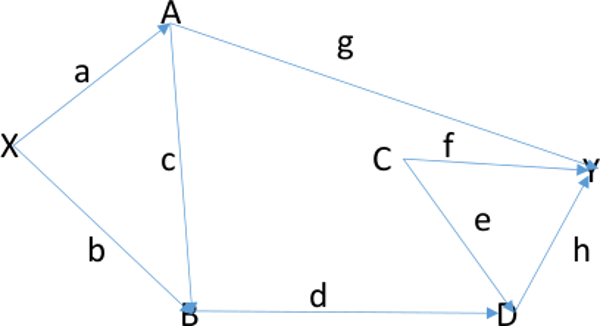
**

**Figure S2.** Diagram associated with effect decomposition.


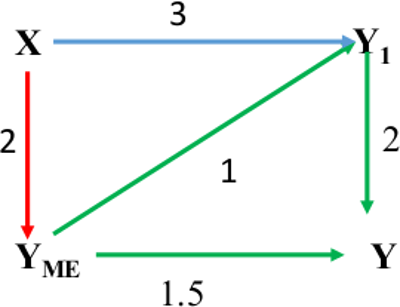


**Figure S3.** Diagram of a simulation example for illustrating equation (12).

**
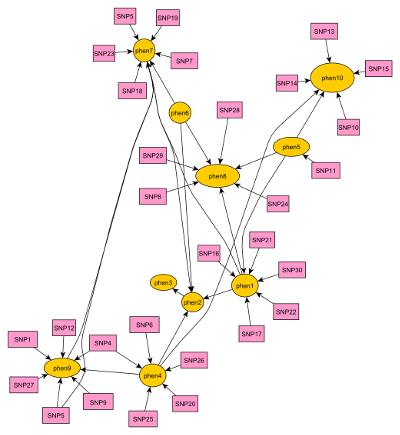
**

**Figure S4.** An example for the simulated genotype-phenotype network. The network consisted of 10 phenotype nodes and 30 genotype (SNP) nodes.


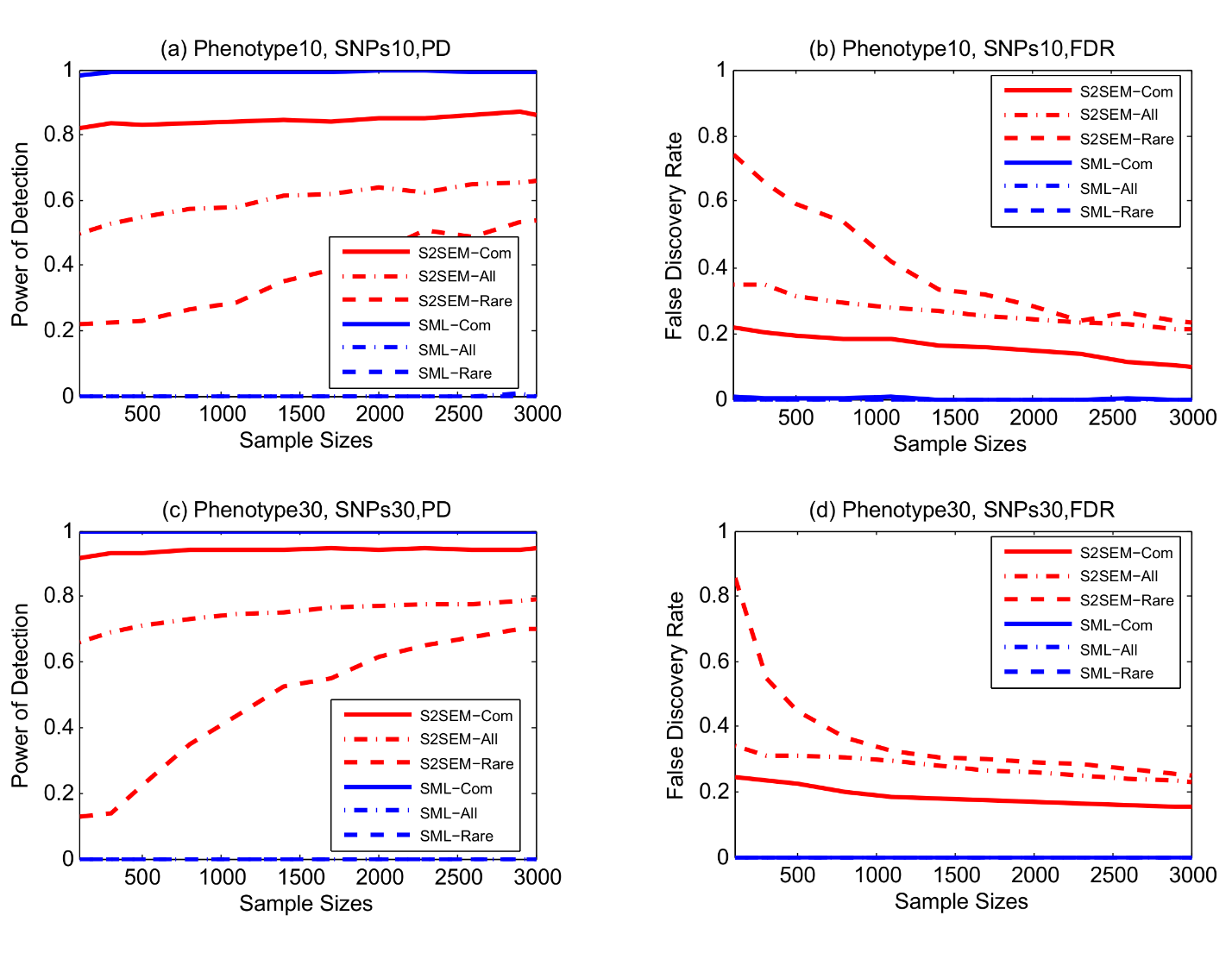


**Figure S5.** Performance of S2SEM and SML for phenotype network inference. The power and FDR of the two methods for inference of phenotype networks when the phenotype and genotype number is 10 and 30 respectively.


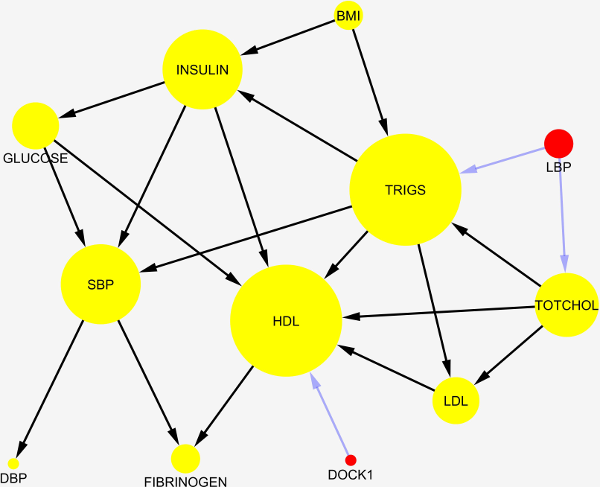


**Figure S6.** A genotype-phenotype network consisted of 2 genes that were reported to be associated with phenotypes in the analysis or other CVD related phenotypes in the literatures and 10 phenotypes (one isolated phenotype didn’t appear) estimated using QTLnet method. The nodes in yellow color represented the phenotypes, the nodes in the red color represented genes, the black arrows indicated the causal relations between phenotypes and the blue arrows indicted the contribution of the gene to one phenotype.
